# The effect of cognitive training on the brain’s local connectivity organization in healthy older adults

**DOI:** 10.1038/s41598-019-45463-x

**Published:** 2019-06-21

**Authors:** Lifu Deng, Yan Cheng, Xinyi Cao, Wei Feng, Hong Zhu, Lijuan Jiang, Wenyuan Wu, Shanbao Tong, Junfeng Sun, Chunbo Li

**Affiliations:** 1 School of Biomedical Engineering, Shanghai Jiao Tong University, Shanghai, China; 2 Shanghai Key Laboratory of Psychotic Disorders, Shanghai Mental Health Centre, Shanghai Jiao Tong University School of Medicine, Shanghai, China; 3 Department of Psychiatry, Tongji Hospital, Tongji University School of Medicine, Shanghai, China; 4 Institute of Psychology and Behavioural Science, Shanghai Jiao Tong University, Shanghai, China; 5 Brain Science and Technology Research Centre, Shanghai Jiao Tong University, Shanghai, China; 6 Centre for Excellence in Brain Science and Intelligence Technology (CEBSIT), Chinese Academy of Science, Shanghai, China; 7 Clinical Neurocognitive Research Centre, Shanghai Mental Health Centre, Shanghai Jiao Tong University School of Medicine, Shanghai, China

**Keywords:** Cognitive ageing, Human behaviour

## Abstract

Cognitive training has been shown effective in improving the cognitive function of older adults. While training related plasticity of the brain has been observed at different levels, it is still open to exploration whether local functional connectivity (FC) may be affected by training. Here, we examined the neuroimaging data from a previous randomized-controlled double-blinded behavioural study, in which healthy older adults participated in a 3-month cognitive training program. Resting-state fMRI was acquired at baseline and one year after training. The local FC in the brain was estimated using the regional homogeneity (ReHo), and the high ReHo clusters (HRCs) were extracted to quantify the level of local FC integration. Results showed that: (i) HRCs exhibited a power-law size distribution; (ii) local FC were less integrated in older participants than in younger participants; (iii) local FC in older participants of the training group became more integrated after training than the control group; (iv) the baseline local FC integration was positively correlated with educational level. These results indicated a training-related alteration in local FC.

## Introduction

Aging is accompanied by a decline in cognitive functions, which affects the life quality of older adults^1–3^. Developing approaches to mitigate or reverse this cognitive decline is a topic that draws attention from both the public and the research community. High levels of mental activity have a positive effect on cognitive ability^4^, which inspires research in designing standardized, repeatable trainings that aim to augment specific cognitive domains or improve general cognitive ability. Different aspects of cognitive training, such as strategy, efficacy, training specificity and transfer, and the maintenance of training outcome, have been investigated^5–8^. In general, cognitive training has turned out to be a promising approach to improving cognitive function in aging populations.

In addition to the cognitive outcome of training, the neural mechanism of cognitive training also draws much attention^3^. The brain’s plasticity under cognitive training has been observed in different modalities, such as metabolism^9^, grey matter structure^10–12^ and white matter integrity^13–15^. Neural plasticity was also reflected by signatures of functional brain activities, including EEG-derived biomarkers^16–18^, fMRI-based resting-state functional connectivity (FC)^19–22^, and task activation^23–26^. Besides specifically-designed training, other forms of training, such as training for sport^27^ and music^28^, may also lead to neural plasticity. In short, these researches suggested that training induces plasticity in the brain at different levels.

How cognitive training would influence short-distance, local-scale FC is still unclear. Local FC, examined using the regional homogeneity (ReHo) method, is associated with a broad category of neurophysiological alterations^29–33^. Specifically, altered local FC is observed in aging and cognitive decline^34–36^. In addition, a recent study on healthy aging participants showed that ReHo strongly correlates with glucose metabolism as measured by positron emission tomography (FDG-PET)^37^. This study further addressed the importance of the regions with high local FC, known as the functional ‘hubs’^38,39^ in the local FC network, and linked the fMRI-based non-invasive ReHo method to the field of aging and cognitive decline, where PET has been a golden standard. These observations call for further attention to the role and significance of local FC in aging and cognitive training.

The duration of neuro-plasticity after training is another concern regarding the efficacy of cognitive training. It has been reported that structural changes after training is non-static and may gradually recede^10^. However, the long-term functional alteration after training has rarely been described. Besides, higher-dosage trainings (3 months or more) with neuro-image acquisition in the follow-up are still rare in the literature. In our current research, we aimed to investigate the above issues by examining the fMRI data from normal older adults that participated in a 3-month cognitive training program consisting of 24 sessions. These participants were a subset of a previously-published, larger-sample study with identical training design, in which sustained improvements in behavioural outcome were observed in a one-year post-training period^8^. The older participants in our study took fMRI scanning and cognitive measurements at baseline and one year after training, which allowed us to explore alteration in brain functional activity over an extended period.

Based on the previous findings, in this study, we hypothesized that the organization of local FC hubs might be affected by the process of aging. We also hypothesize that local FC hubs may be sensitive to cognitive training. Furthermore, if training effect exists, we predict that local FC should also have association with traits related to cognitive function. In order to test these hypotheses, we focused on brain regions that are in the highest percentile of ReHo distribution, i.e., the local ‘hubs’. These high ReHo regions formed spatially separated clusters, referred to as high ReHo clusters (HRCs) in the following text. Under the framework of brain graph analysis^40^, an HRC resembles a connected component in a local FC network with a given connection sparsity. The level of integration of HRCs may reflect the efficiency of the local FC network, as larger HRCs facilitate information exchange (in the form of coupled BOLD activity) across broader regions. For every participant, such local FC integration can be quantified as the average size of the HRCs, or inversely, as the number of HRCs. We investigated the difference in local FC integration between the older participants and another group of younger participants (aged from 25 to 55), and further examined the effect of training on the local FC integration in older participants. We then examined the association between educational level and local FC integration, and the association between cognitive outcome of the training and alteration of local FC integration. In short, our study indicated the effect of cognitive training on the human brain’s local FC network. This study also indicated that the neuro-physiological relevance of the organization of local functional network may deserve further attention.

## Materials and Methods

### Participants

The participants enrolled in the present study are in the MRI sub-sample of our previous study^8^, in which healthy older adults were recruited from three community centres near Tongji Hospital in Shanghai via a dispatched notice/broadcasting by the local community service. Recruitment of MRI sub-sample was performed by sending advertisement to all participants in the previous study. Participants who responded to the advertisement were assessed for MRI eligibility before being included into the MRI sub-sample (See also^41^). The study was registered with the Chinese Clinical Trial Registry (http://www.chictr.org.cn) (Registration Number: ChiCTR-TRC-08000732).

Participants were admitted to the study according to the following inclusion criteria: (1) normal functional capacity; (2) independent living in the community; (3) age ranging from 65 to 75 years; (4) educational level more than one year; (5) no dysfunction in hearing, vision, or communication ability; (6) the Chinese version of the Mini-Mental State Examination (MMSE) score of 19 or above (the minimum MMSE score was 22 in this dataset; the lower normal cut-off point was due to the lower average educational level in Chinse older adults; in this sub-sample, all participants have MMSE scores above the education-specific cut-off^42^); and (7) no physical disease, psychotic disorder, obvious cognitive decline, diagnosis of Alzheimer’s disease, brain tumour, or other serious neurological disorders.

Participants were randomly assigned into three groups: the multi-domain training group, the single-domain training group, and the control group, and they underwent cognitive measurements and fMRI scanning at baseline and at one year after training. Eleven participants (four in the multi-domain group, four in the single domain group, and three in the control group) were excluded from our analysis due to missing follow-up cognitive assessments or MRI sessions. This study was designed under guidelines of the Declaration of Helsinki, and was reviewed and approved by the Human Research Ethics Board of Tongji Hospital. All participants gave written informed consent.

In addition, this study incorporated the data from 21 healthy younger adults in another study to serve as a contrast group to the older adult participants. These younger participants underwent a single-session fMRI scanning in the same centre using identical scanner and parameters, and were screened for neurological and psychiatric diseases. The younger participants ranged from 25 to 55 in age (38.53 ± 10.38 years). Eight participants were males. Participants had an educational level of 12.38 ± 3.41 years. Since the educational level in younger adults was higher (p = 0.045, Wilcoxon test), we have corrected this factor in our analysis in order to avoid confounding effects.

### Cognitive training and neuropsychological tests

The multi-domain and single-domain training group received 24 sessions of cognitive training twice a week in Tongji Hospital, over a period of 12 weeks. Each session lasted for 60 minutes in small group setting (~15 participants per group), in which the trainer taught specific cognitive techniques, and instructed the participants to practice these techniques. The multi-domain training targeted memory, reasoning, problem-solving strategies, visual-spatial map reading skills, handicrafts, and physical exercise. The single-domain training targeted reasoning skills. The control group served as a match for the social contact associated with cognitive training. Participants from the two training groups and the control group attended a lecture about healthy living every two months. Six months after the end of the initial 24 training sessions, booster training was randomly assigned to participants in each training group to reinforce the initial training. The booster training comprised three 60-minute sessions, with one session per month for three months. During booster sessions, previously practiced contents were reviewed. More training details can be seen in^8^.

To evaluate the effects of cognitive training, cognitive assessments were carried out at baseline and at one-year post-test by trained research personnel who did not know about the group assignment of the participants, according to the directions in^43^. The Repeatable Battery for the Assessment of Neuropsychological Status (RBANS)^43^ was administered, which consists of five index scores (immediate memory, visuospatial/constructional, language, attention, and delayed memory) and one total score. Participants received the Form A of the RBANS modified for Chinese participants, which has been shown to have good reliability and validity in previous studies^44,45^. Besides RBANS, colour word Stroop test^46^, visual reasoning test from the WHO Neuropsychological Battery of Cognitive Assessment Instruments (WHO-BCAI), and trail making test^47^ were also administered. Since the RBANS and visual reasoning test assessments showed significant training effects in our previous study^8^, we will present the results of RBANS and visual reasoning test of the current MRI sub-sample in the following Results section.

### Data acquisition and pre-processing

Older participants were scanned in a Siemens (Erlangen, Germany) 3T MRI scanner at baseline and at one year after training completion at East China Normal University in Shanghai, China. The younger contrast group participants were scanned once in the same imaging centre. To minimize head motion, foam pads were used to fix the participants’ heads. High-resolution T1-weighted images were acquired using a magnetization-prepared, rapid gradient-echo sequence, generating 160 slices (repetition time (TR) = 1900 ms, echo time (TE) = 3.43 ms, flip angle (FA) = 90 degrees, field of view (FOV) = 256 × 256 mm^2^, matrix size = 256 × 256, voxel size = 1 × 1 × 1 mm^3^, slice thickness = 1 mm (no gap)). Resting state functional images were acquired using a single-shot, gradient-recalled echo planar imaging sequence (TR = 2000 ms, TE = 25 ms, flip angle = 90 degrees, FOV = 240 × 240 mm^2^, matrix = 64 × 64, slice thickness = 5 mm (no gap), 32 slices per volume). All participants underwent a 310 sec scanning, yielding 155 volumes in total. The participants were instructed to relax and keep their eyes closed, not to think of anything in particular, and not to fall asleep.

The fMRI data were pre-processed in standard procedure using SPM 8 and DPARSF pipeline^48^. The first four frames were removed, and raw images were slice-timing corrected. All slices were realigned to eliminate motion artefacts, and no participant was excluded due to excessive head motion (more than 1.5 mm and 1.5°). Then the functional images were co-registered to the T1 image. After that, the T1 images were normalized by registering them to the standard MNI152 template using affine transform, and the normalized T1 images were segmented into grey matter, white matter, and cerebrospinal fluid by using a unified segmentation algorithm^49^. The co-registered functional images were also registered into standard space and were resampled into a resolution of 3 × 3 × 3 mm^3^. The BOLD signal was then de-trended and band-pass filtered (0.01 Hz–0.08 Hz). The six head motion parameters were also regressed out from the BOLD signal. In addition, the frame-wise displacement (FD) was also calculated to quantify head motion for each participant^50^, and was controlled as a potential confounding factor (although we found no correlation between FD and HRC measurements). Spatial smoothing was not performed, as this step would have affected the result of local FC measurement and reduced its reliability^51,52^.

### Regional homogeneity (ReHo) and the high-reho clusters (HRCs)

**Figure 1:**
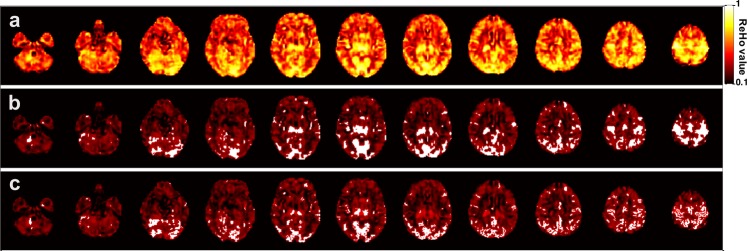
Demonstration of high-ReHo cluster (HRC) from a typical participant. (**a**) Whole-brain ReHo map of a typical participant. (**b**) High ReHo clusters (HRCs), constituted by the top 10% voxels among all voxels, are marked with white colour, and the background brain regions are dimmed in order to highlight the HRCs. (**c**) The shape of HRCs in (**b**) is further constrained by the shape of grey matter.

The local FC was calculated using ReHo algorithm^53^:

$${Re}Ho=\frac{{\sum }_{i=1}^{N}{R}_{i}^{2}-N{\bar{R}}^{2}}{\frac{1}{12}{K}^{2}({N}^{3}-N)},$$

where *N* denotes the length of the time series, *K* = 27 is the size of the voxel cluster containing 3 × 3 × 3 adjacent voxels, *R*_*i*_ denotes the summation of the rankings of BOLD signal amplitude of all *K* voxels at the *i*th time point, and $\bar{R}$ is the mean of *R*_*i*_. The ReHo in the whole brain was mapped for each participant, and the result was then resampled to 2 × 2 × 2 mm^3^ for further analysis and for visualization (Fig. 1a).

For the ReHo maps of each participant, brain regions with the highest ReHo (e.g., top 5%, top 10%, and top 15% among all voxels inside the brain) were extracted. These regions formed spatially isolated clusters (i.e., HRCs) that delineated all the local FC hot-spots in the brain (Fig. 1b). One concern of analysing HRCs is that two or more HRCs may appear as a single cluster due to the cortical folding patterns. To resolve this issue, the shapes of the clusters were further constrained by the shape of grey matter of each individual (produced in the unified tissue segmentation during the pre-processing), ensuring that each high-ReHo cluster was truly formed as a connected region in anatomy (Fig. 1c). On the other hand, we noticed that the geometry of grey matter could also be associated with age, which might complicate the analysis of the HRC. Therefore, we also examined HRCs without applying anatomical constraint, and the results were largely consistent (Supplementary Fig. 1).

### Statistical analysis

The size of one HRC can be regarded as spatially integrated local FC network. Thus, a whole-brain quantification of local FC integration for one participant can be represented as the average size of all HRCs, or, inversely, by the number of HRCs inside the brain. We found that the size distribution function of HRCs showed a power law tail, resembling a so-called scale-free feature. To visualize this distribution, the logarithmic binning technique^54^ was employed, and the probability distribution was plotted in log-log coordinate. We also estimated the power law exponent of the distribution for all the participants by performing linear regression on the power law tail in log-log coordinate.

Two-sample t-test was used to compare HRC in baseline older participants and younger participants. Before examining the training-versus-control effect, we controlled for attendance and excluded participants in the training groups with low attendance rate (n = 9, attendance = 11.11 ± 5.42 sessions, cut-off criterion = 19 total sessions or less, see Supplementary Table 1 for detail). To investigate the training effect, we performed ANOVA on the before-after differences in HRC number and mean HRC size across three groups (multi-domain/single-domain/control). Within-participant changes in HRC number and size were then examined with paired t-test. We also investigated training effect by performing two-sample t-test between the control group and the merged training group (i.e., multi-domain group + single-domain group). This yielded a larger statistical power, which is helpful in this exploratory study with limited participants in each group. The two training groups were matched in demography, HRC number, HRC size, and behavioural score before and after training, which justified the merging of the two training groups as an exploratory approach. The results with two training groups merged were presented in the main text, and the results with multi-domain group and single-domain group separately analysed were presented in Supplementary Fig. 2 for maintaining the rigor of our analysis. Results from these two version of analyses are largely convergent.

All t-tests were two-sided unless specified. Confounding factors such as education, gender, and FD were regressed out from the data using linear model before further between-group and within-group analyses. The Kolmogorov-Smirnov test was carried out to test normality of data, and non-parametric tests were carried out when the data did not satisfy normality. False discovery rate (FDR) correction^55^ was performed in the case of multiple comparisons.

## Results

### Demographic information and the effect of cognitive training

Table 1 listed the demographic information of the participants with complete behavioural measurements and MRI data in the three groups. One-way ANOVA and Kruskal-Wallis tests suggested that the three groups were matched in age, gender, education, MMSE score, and head motion (i.e., FD) at before-training baseline. Besides, no significant within-group difference in FD was found between baseline and one-year after training.

**Table 1:** Demographic information of the older adult participants at baseline.

	Multi-domain	Single-domain	Control	p-value
Age (years)	72.39 ± 3.43	70.26 ± 3.90	70.85 ± 4.05	0.78
Gender (male)	18 (13)	18 (9)	14 (9)	0.23
Years of Education	11.11 ± 4.25	8.37 ± 4.00	10.09 ± 3.34	0.45
Baseline MMSE	27.57 ± 2.57	27.91 ± 1.77	28.17 ± 1.94	0.33
FD, Baseline	0.18 ± 0.087	0.18 ± 0.073	0.15 ± 0.081	0.51
FD, 1-yr after	0.19 ± 0.089	0.22 ± 0.10	0.17 ± 0.072	0.26

The educational level in younger adults was higher than that in the older adult (p = 0.045, Wilcoxon test), and this factor was corrected in our following analysis to eliminate confounding effects. No significant difference was found between FD in younger participants and that of older participants in baseline.

The influence of training on different cognitive measures was tested by one-way ANOVA (Table 2). Training significantly enhanced the RBANS delayed memory score (F(2,43) = 3.67, p = 0.03, uncorrected) and marginally influenced the score of language (F(2,43) = 2.87, p = 0.07, uncorrected). However, these training effects did not survive after correction of multiple comparisons. No training effects were observed in RBANS total score (F(2,43) = 1.96, p = 0.15) and visual reasoning test (F(2,43) = 2.68, p = 0.11), although the statistics hinted a trend of higher improvements in the two training groups. Note that these four cognitive measurements showed significant training effect in the previous large-sample study (Cheng *et al*.^8^). No group effect was found at baseline. In general, the cognitive measures in the MRI sub-sample were consistent with the previous large-sample study, but with lower statistic power.

**Table 2:** The RBANS total score and sub-category scores (mean ± SD) of the older adult participants at baseline and one year after training.

	Multi-domain		Single-domain		Control		*P* ^Δ^	*P* ^baseline^
	Baseline	One-year after	Baseline	One-year after	Baseline	One-year after		
RBANS total score	93.17 ± 16.10	106.94 ± 12.90	92.44 ± 14.06	102.5 ± 16.29	93.07 ± 15.00	99.57 ± 13.75	0.15	0.98
-Immediate memory	86.22 ± 16.45	103.17 ± 23.35	87.39 ± 15.95	101.61 ± 18.67	84.00 ± 14.34	100.5 ± 14.33	0.86	0.81
-Visuospatial	106.78 ± 12.86	103.9 ± 11.56	99.39 ± 11.02	103.50 ± 17.24	106.86 ± 19.05	103.00 ± 16.81	0.13	0.14
-Language	92.44 ± 12.92	101.00 ± 8.39	94.94 ± 9.98	99.11 ± 8.47	93.29 ± 7.12	95.86 ± 5.86	0.07(+)	0.78
-Attention	90.67 ± 19.39	94.94 ± 15.17	92.00 ± 15.97	94.94 ± 18.57	91.5 ± 15.25	88.93 ± 15.24	0.36	0.97
-Delayed memory	98.28 ± 18.86	118.50 ± 12.41	98.94 ± 19.81	109.56 ± 19.79	99.00 ± 13.13	110.21 ± 14.80	0.03(*)	0.99
Visual reasoning test	5.78 ± 1.35	6.56 ± 1.89	5.22 ± 2.05	6.17 ± 2.48	5.86 ± 2.74	5.93 ± 2.46	0.11	0.35

***P***^**Δ**^: One-way ANOVAs across groups with differences between one year after training and baseline (before training) in cognitive measures as dependent variables, and scores of the psychological measures at baseline, age, gender, and education years as covariates. ***P***^***baseline***^: One-way ANOVAs across groups with scores of the measures at baseline as dependent variables, and age, gender, and education years as covariates. (^+^p < 0.1, *p < 0.05). As a reference, in our previous larger-sample behaviour study, RBANS total score, immediate memory, language, delayed memory, and visual reasoning test showed significant training effects (Cheng *et al*.^8^).

### HRC overview: number, average size, distribution, and age difference

**Figure 2:**
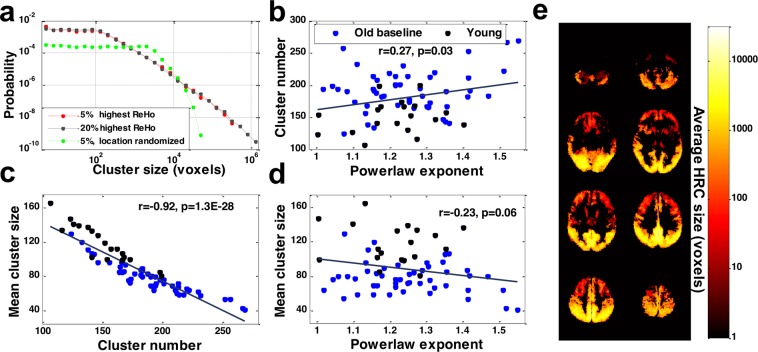
The quantitative profile of HRCs. (**a**) The size distribution of HRCs at the group level (all the older participants at baseline). HRCs were extracted with two different thresholds (5% and 20% highest ReHo voxels) and with the anatomic constraint of grey matter. The size distribution lost its power-law property when ReHo images were randomized (green line). (**b**–**d**) The relationships between cluster number, mean cluster size, and the power law exponent of the HRC size distribution (black dots: younger participants; light blue dots: older participants at baseline). (**e**) The average HRC size in space. The HRCs in (**b**–**e**) were composed of the highest 10% ReHo voxels for each participant and were constrained by grey matter geometry.

The size of HRC manifested a power law-like distribution after a turning point (approximately 100 voxels). This long tail feature remained stable across a range of ReHo thresholds (5–20%). Note that the long tail property of HRC size distribution disappeared when all the voxels in a ReHo image were randomly shuffled (Fig. 2a), which further indicates the occurrence of local FC hubs are not occasional. We then calculated the power law exponent of the HRC size distribution, the number of HRCs, and the mean size of HRCs for all participants (i.e., all older participants at baseline, and all younger participants). Pearson’s correlation analysis was carried out to examine the relationship between them. The power law exponent was positively correlated with the number of HRCs (Fig. 2b) and negatively correlated with the mean size of HRC with marginal significance (Fig. 2d) across all participants. The mean HRC size and the number of HRCs were strongly and negatively correlated (Fig. 2c). Note that in these three scatterplots the young participants and the older participants are discernible. We also illustrated the spatial distribution of HRC size in Fig. 2e, which was obtained by assigning each voxel the size of the HRC that it belonged to, and averaging the size across all participants. Larger HRCs mainly appeared in occipital lobes and post cingulate cortex, and the average HRC size decreased as the location moved from posterior to anterior.

**Figure 3:**
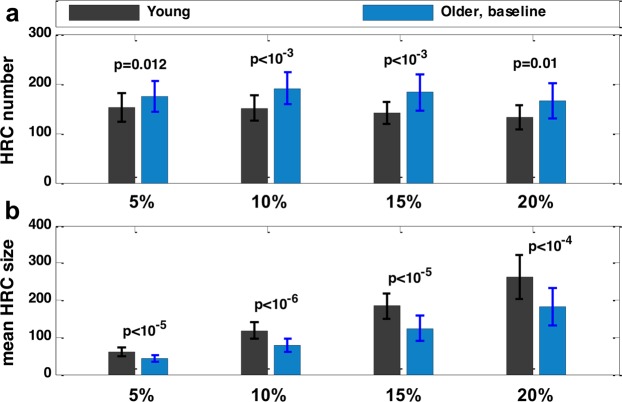
(**a**) HRC number and (**b**) average HRC size of young and older participants. HRC number and average HRC size were calculated under four different ReHo thresholds. The error bars indicate standard deviation. P-values were from two-sample, two-sided t-tests. Potential confounding factors (gender, education, FD) were corrected.

The panels b, c, and d in Fig. 2 hinted at an age-related difference in HRC. In fact, compared with younger participants, older participants at baseline showed a significantly less integrated local FC pattern, in terms of HRC number (Fig. 3a) and the mean HRC size (Fig. 3b). More importantly, the differences between younger and older participants at baseline were significant across different thresholds used for HRC extraction, which suggests HRC’s good consistency and parameter-insensitivity in revealing the between-group differences. Such age-related difference was also observed when the geometry of grey matter was not taken into consideration (Supplementary Fig. 1).

### Training effects on high-ReHo clusters

**Figure 4:**
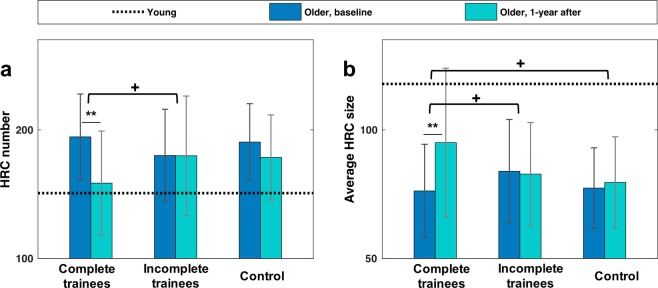
Training effect on HRC. (**a**) HRC number of the complete trainees, incomplete trainees, and control participants. (**b**) Mean HRC size of the complete trainees, incomplete trainees, and control participants. The error bars indicate standard deviation. Dashed lines indicate the average values of these two measurements in younger participants. Solid lines indicate within-group comparison, while horizontal square brackets indicate between-group comparison (**p < 0.01, ^+^p < 0.1). HRCs in this figure were defined as the 10% highest ReHo values, and were constrained by grey matter shape. No between-group difference in HRC number or mean HRC size was found at baseline. Potential confounding factors (gender, education, FD) were corrected.

We examined whether cognitive training was associated with the alteration of local FC integration before and after training. The results of training effects on HRC are shown in Fig. 4. No significant between-group difference was found at baseline. Older participants who completed the training in the two training groups (i.e., attended sessions >19) exhibited significant before-after alterations in HRC number (t(26) = −3.40, p = 0.002) and mean HRC size (t(26) = 3.17, p = 0.004), showing a more integrated local FC one year after training, which was closer to the configuration in younger adults. On the contrary, the control group and the incomplete trainees exhibited no significant change in these two measurements. Crucially, the before-after alteration in HRC number showed a greater trend in complete trainees than in incomplete trainees (t(34) = 1.74, p = 0.090). The before-after alteration in average HRC size also differed between complete and incomplete trainees (t(34) = 1.77, p = 0.085), and between complete trainees and control participants (t(39) = 1.84, p = 0.074) with marginal significance. Such between-group difference suggested an association between cognitive training and the change of local FC integration.

**Figure 5:**
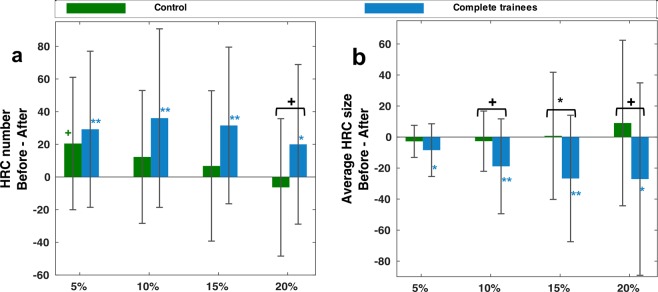
The before-after difference of (**a**) HRC number and (**b**) average HRC size in four different ReHo thresholds. The error bars indicate standard deviation. Horizontal square brackets in black indicate between-group comparison, while markers adjacent to the bars indicate within-group comparison (**p < 0.01, *p < 0.05, ^+^p < 0.1).

To test the robustness and reliability of the result, we examined training effect on HRC obtained at four different ReHo thresholds. The completers in the training group consistently showed significant increase in average HRC size and decrease in HRC number at all the four threshold levels; on the contrary, the older adult control group showed no significant within-group difference in HRC size or in HRC number between the baseline and the one-year post-test (Fig. 5). Importantly, a marginally significant training main effect (F(1,36) = 3.77, p = 0.06) was revealed by a *Group* (complete trainee/control) by *Threshold* (5/10/15/20%) ANOVA for the difference in average HRC size (*Threshold* as the repeated measure). Subsequent between-group comparison revealed marginally significant differences in before-after changes in HRC number at the 20% threshold level (t(39) = 1.72, p = 0.094), and in before-after changes in average HRC size at 10%, 15%, and 20% threshold levels (t(39) = 1.84/2.08/1.86, p = 0.074/0.044/0.071, respectively), suggesting that the local FC became more integrated after training. In addition, with the variation of threshold, the training group and the control group showed distinguishable trends in the before-after difference in average HRC size, as revealed by a marginally significant Group * Threshold interaction effect (F(3,117) = 2.46, p = 0.067).

We also examined the multi-domain group and single-domain group separately, and the results are summarized in Supplementary Fig. 2. Significant before-after changes in HRC number and average HRC size were observed within multi-domain completers, and the results were robust across different ReHo thresholds. Importantly, the before-after difference in average HRC size was significantly different between multi-domain completers and control group at 10%, 15%, and 20% ReHo thresholds. Meanwhile, the before-after difference in HRC number was significantly different between these two groups at 20% ReHo threshold, and marginally different at 10% and 15% ReHo thresholds. The before-after change of HRC was less significant within single-domain completers, but no significant difference was found between the two training groups. ANOVA revealed marginal significance of group effect on before-after change in average HRC size, at 10% and 15% ReHo thresholds (F(2,40) = 2.73, p = 0.078, and F(2,40) = 2.63, p = 0.085, respectively).

**Figure 6:**
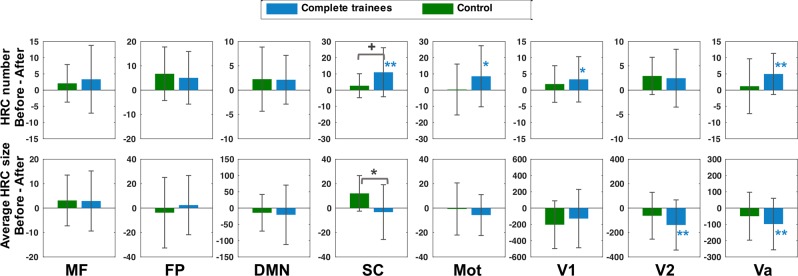
Training effects on HRCs in different functional networks. Here, the HRCs were extracted at the threshold of the 10% highest ReHo, and were constrained by the shape of grey matter. The error bars indicate standard deviation. Horizontal square brackets in black indicate between-group comparison (FDR un-corrected), while markers adjacent to the bars indicate within-group comparison (FDR corrected). (**p < 0.01, *p < 0.05, ^+^p < 0.1).

We further examined the before-after changes in HRCs across different functional regions between control participants and training participants. We employed a template^56^ that parcellated the brain into eight functional networks, namely, the medial frontal network (MF), the frontoparietal network (FP), the default mode network (DMN), the subcortical-cerebellum network (SC), the motor network (Mot), the visual I network (V1), the visual II network (V2), and the visual association network (Va). For computing the network-wise HRC number, an HRC was labelled by a functional network if it overlapped with that functional network. The overlapped volumes were then taken into calculation of the average HRC size in that functional network. The SC network showed the strongest training-vs. control effect. At the 10% ReHo threshold, change in the HRC number in the SC network differed with marginal significance between the complete trainees and the control participants, and in the same network, the change in average HRC size differed significantly between the two groups (Fig. 6). At 15% and 20% threshold level, the differences in HRC number and average HRC size both consistently showed significant difference (p < 0.05) between the two groups. However, the significant differences between complete trainees and control participants became insignificant after FDR corrections.

### The correlation between HRCs and education

Linkages between cognitive score and the characteristics of HRC, if they exist, may offer a possible interpretation of the mechanisms of cognitive training. We performed a correlation analysis between the baseline RBANS and the baseline HRC characteristics (i.e., average size and number), as well as between the improvement in RBANS and the before-after difference in HRC. However, neither of the two analyses exhibited significant correlation. We also explored the relationship between RBANS sub-scores and HRCs in the eight functional networks respectively. No significant correlation was found after FDR correction.

**Figure 7:**
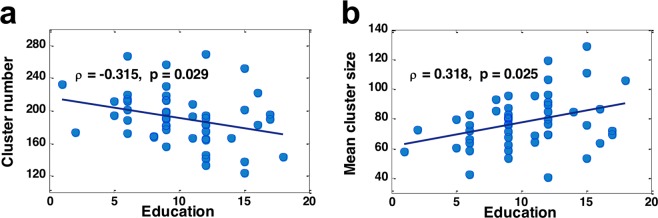
(**a**) The relationship between HRC number and years of education. (**b**) The relationship between mean HRC size and years of education. In this figure, HRCs here were defined as the 10% highest ReHo voxels for each individual and were constrained by grey matter geometry.

Although HRC changes were uncorrelated with training outcome, we observed that the years of education significantly contributed to the cross-individual differences in local FC integration at baseline. Educational level was negatively correlated to HRC number (ρ = −0.315, p = 0.029) (Fig. 7a), and positively correlated to mean HRC size(ρ = −0.318, p = 0.025) (Fig. 7b). These trends remained consistent at multiple ReHo threshold levels that were used in HRC extraction. The results remained consistent in the presence and absence of anatomic constraint. We also found that the HRC number was negatively correlated with educational level in the FP network (p = 0.012) and the SC network (p = 0.043), but the significance did not survive FDR correction.

## Discussion

In this study, we observed a training-related increase in the integration of local FC. Together with the observation that local FC in older adults was less integrated compared with that of younger adults, our results suggested that cognitive training may be able to shape the local FC into a profile more similar to that in younger participants. A more integrated local FC pattern may reflect higher efficiency of the local FC network and may facilitate cognitive function, as is supported by the fact that HRC integration was associated with exposure to education. These results may provide new perspectives in understanding the neurological effects of cognitive training.

### The scale-free local FC hubs

Brains exhibit functional connections in a wide spectrum of spatial distance. While long distance connectivity can be comparable to the highway networks connecting distant areas, local connectivity may be likened to the network of streets that aggregate adjacent areas into a city. In our study, the local FC network was examined by thresholding the ReHo map. This technique, although not a direct mapping of the local FC network, clearly presents the connected components in the local FC network, which has a power-law distribution. Interestingly, the size distribution of the local FC hubs (i.e., HRCs) is similar to the city-size distribution, as it also showed a long tail, power-law feature (a.k.a. scale-free organization).

Power-law distribution, as a signature of complexity, is observed in different forms of complex networks. In neural system, such signature has been found in the macroscopic structural and functional brain networks^57–59^ and in the microscopic neuronal network^60^ as well. In this study, we reported the scale-freeness in the regional FC network, which is made up by local functional connections that are several millimetres in length. The biological significance of such scale-free organization was further underscored by the fact that the power-law distribution was absent in randomized ReHo maps, suggesting that a scale-free organization of the local FC should be an intrinsic feature of brain rather than a random organization. In addition, it is worth noting that the strength of local FC is also associated with temporal complexity of BOLD activity, as measured by the scale-free feature in the frequency spectrum of BOLD signal^61^, which further suggests the functional significance of HRCs in local FC network.

### The long-term training effect in local FC

In the present study, we aimed to explore long lasting neural effects of cognitive training. Previous studies^10,11^ have shown neural plasticity immediately after training, which then showed a trend of recession during a three-month follow-up period. Therefore, the sustainability of neural plasticity is worth examining in an extended period of time. In our study, the training-related plasticity was observed after one year, which is in parallel with the sustained behavioural outcome observed in our previous study^8^. The behavioural results in the current sub-sample also showed moderately good consistency with the previous large-sample study, although the statistic power was limited by the size of current fMRI sub-sample.

Such long-term plasticity may be due to the larger training dosage in our study, which is supported by the fact that before-after difference was only observed in participants receiving adequate training shown in Fig. 4. The potential existence of long lasting neural effects of cognitive training is also indicated by the correlation between education and HRC integrity, as the education that the participants had received (although happened several decades ago) correlated with the integrity of the HRCs. There is notable similarity between cognitive training and education in general, as both require a high level of cognitive engagement. In fact, a previous study^62^ has demonstrated the connection between education and brain volume.

### Local FC integration and cognitive function

We noted that although HRCs changed in size and number after training, these changes did not correlate with the improvement of the RBANS scores, nor did the baseline HRC number or size correlate with the baseline RBANS score. Such dissociation between cognitive function and the neuroimaging markers may be due to the hiatus between the two fMRI acquisitions, or a possible learning effect resulting in cross-session contamination (Park and Bischof, 2013)^63^ in the RBANS assessment.

It is worth noting that we observed significant training effect in the subcortical-cerebellum region which was defined by a published atlas^56^. Within this region, the basal ganglia system has been shown to play a key role in cognitive skill learning^64–66^. However, caution should be taken in interpreting this result, since the significance did not survive FDR correction, and no correlation between cognitive outcome and HRCs in the subcortical-cerebellum region was found. In short, further study is needed to clarify whether the change in local FC accounts for the behavioural outcome of the cognitive training.

### Limitations

Some limitations in this study may be improved in the future. First, caution should be taken when interpreting the difference between younger participants and older participants. Although we corrected demographic differences (such as education) that may bias the result, it should be noted that other factors such as nutrition and culture (absent in our data) might differ between the two age groups as well.

Secondly, the follow-up fMRI acquisition took place one year after the training ended. While this allowed investigation of the long-term effect of cognitive training, it is not clear whether local FC was affected during the training process. It might be possible that, the brain was shaped during the period after training completion, due to factors such as the acquisition of new cognitive strategies or change in life styles. Thus, future study is needed to address the temporal dynamics of brain plasticity with multiple fMRI data acquisition across a longer time span.

Thirdly, the relatively small sample size has limited the interpretation of our results. When examining the multi-domain group and the single-domain group separately (Supplementary Fig. 2), the result indicated that cognitive training may be associated with more integrated local FC, but the statistical power of the group effect was weaker. Merging the two training groups into one increased the statistical power and revealed a significant training effect, but resulted in the difficulty in interpreting such effect, because the two groups might be heterogeneous. Although HRC number, HRC size, and behavioural score were matched for the two groups, the results in Supplementary Fig. 2 seemed to suggest that multi-domain training has a greater impact on local FC integration. Therefore, larger sample size may help confirm the training effect and clarify the differences between different training paradigms in future studies.

## Conclusion

We observed an age-related difference in local FC organization, and a long-term training effect on the local FC organization in a sample of healthy older adults. Furthermore, the integration of local FC was associated with the participants’ educational level. These results suggested that cognitive training in older adults may be related to the reorganization of local FC. The HRC method may serve as a sensitive biomarker reflecting functional reorganization in the brain, and a new perspective in probing into the brain as a complex system.

## Supplementary information

Supplementary Material
