# Supplementary material for: The effect of cognitive training on the brain’s local connectivity organization in healthy older adults
Source: Sci Rep. 2019 Jun 21;9:9033. doi: 10.1038/s41598-019-45463-x (PMC6588690; doi:10.1038/s41598-019-45463-x)

Supplementary Material: ‘The Effect of Cognitive Training on the Brain’s Local Connectivity Organization in Healthy Older Adults’

Lifu Deng[1#], Yan Cheng[2#], Xinyi Cao[2,7], Wei Feng[3], Hong Zhu[1], Lijuan Jiang[2], Wenyuan Wu[3], Shanbao Tong[1,5], Junfeng Sun*[1,5], Chunbo Li[2,4,5,6]*

[1] School of Biomedical Engineering, Shanghai Jiao Tong University, Shanghai, China

[2] Shanghai Key Laboratory of Psychotic Disorders, Shanghai Mental Health Centre, Shanghai Jiao Tong University School of Medicine, Shanghai, China

[3] Department of Psychiatry, Tongji Hospital, Tongji University School of Medicine, Shanghai, China

[4] Institute of Psychology and Behavioural Science, Shanghai Jiao Tong University, Shanghai, China

[5] Brain Science and Technology Research Centre, Shanghai Jiao Tong University, Shanghai, China

[6] Centre for Excellence in Brain Science and Intelligence Technology (CEBSIT), Chinese Academy of Science, China

[7] Clinical Neurocognitive Research Centre, Shanghai Mental Health Centre, Shanghai Jiao Tong University School of Medicine, Shanghai, China

# Equal contribution

* Corresponding author: Junfeng Sun: jfsun@sjtu.edu.cn; Chunbo Li: icb@smhc.org.cn

Supplementary Table 1. Summary of participant attendance. No significant differences in attendance were found for either the initial sessions or the booster sessions. Complete trainees were defined as those who attended more than 19 sessions in total.

	**Multi-domain**	**Single-domain**
**Attendance for Initial 24 Sessions**	18.40±5.71	20.00±6.89
**Booster Sessions**		
Number of Enrolled Subjects	15	12
Attendance	2.48±0.63	2.74±0.64
**Number of Complete Trainees**	12	15
Total Attendance	23.87±2.22	24.99±2.05
**Number of Incomplete Trainees**	6	3
Total Attendance	13.67±3.88	6.00±4.58

Supplementary Figure 1. (a) HRC number and **(b)** mean HRC size under four different ReHo thresholds, without anatomic constraint that limited the HRCs to gray matter regions. The asterisk **(*)** indicates significance level at p<0.05, and the plus **(+)** indicates p<0.1

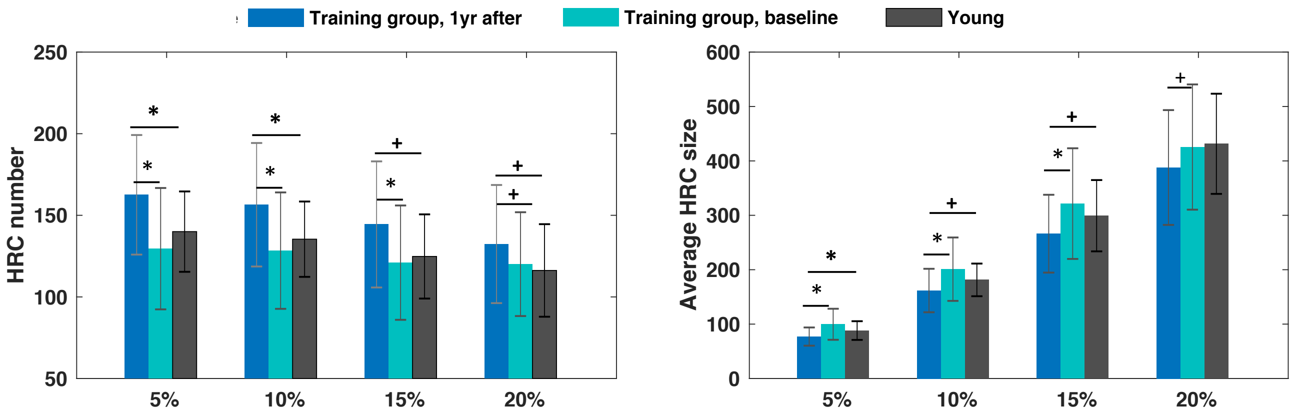

Supplementary Figure 2. A separated examination of the before-after differences for the control group, multi-domain group, and single-domain group, respectively. The differences in HRC number **(a)** and average HRC size **(b)** were examined in four different ReHo thresholds. The asterisk **(*)** indicates significance level at p<0.05, and the plus **(+)** indicates p<0.1.

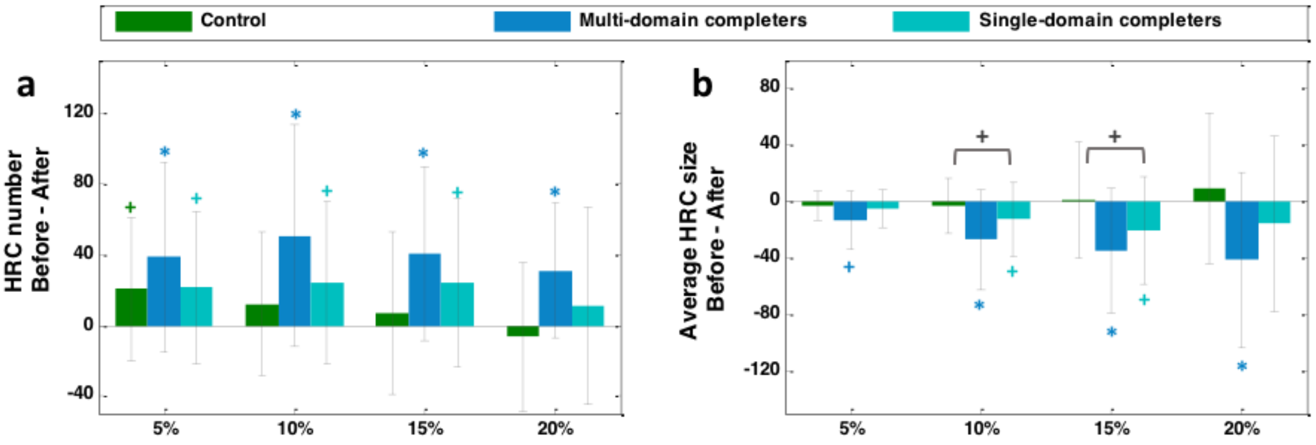

Supplement: Supplementary file 1 — Supplementary Material [file 41598_2019_45463_MOESM1_ESM.pdf]
